# 

**Published:** 1882-09

**Authors:** 


### CONTENTS:

| | PAGE. |
|---|---|
| **ORIGINAL COMMUNICATIONS:** | |
| The Management of an Abortion. By Matthew D. Mann, A. M., M. D. | 49 |
| Something about Oxaluria. By Charles G. Stockton, M. D., | 57 |
| **CLINICAL REPORTS:** | |
| A Case of Excessive Masturbation in the Female, with an Unusual Method of Performance. Reported by J. H. Pryor, M. D., | 62 |
| **SOCIETY REPORTS:** | |
| Meeting of the Buffalo Medical Club, | 66 |
| Buffalo Medical and Surgical Association, | 84 |
| **SELECTIONS:** | |
| Cardiac Symptoms of Chorea, | 87 |
| A New Way to Detect Stone in the Bladder, | 88 |
| Albuminuria as a Symptom of Epileptic Attacks, | 89 |
| Iodoform in Diabetes, | 89 |
| To Cover the Odor of Iodoform, | 90 |
| **EDITORIAL:** | |
| Discussion on the New Code, | 90 |
| Editorial Notes, | 91 |
| **REVIEWS:** | 92 |

